# Unusual Case of Gouty Arthritis of the Second Distal Interphalangeal Joint (Second Toe)

**DOI:** 10.7759/cureus.11405

**Published:** 2020-11-09

**Authors:** Adel H Hegaze, Amre S Hamdi, Abdulraof T Alqrache, Mohamed Hegazy

**Affiliations:** 1 Orthopedics Department, King Abdulaziz University, Faculty of Medicine, Jeddah, SAU; 2 Department of Biochemistry, King Abdulaziz University, Rabigh, SAU; 3 Trauma and Orthopedics, Independent Researcher, Private Sector, Berlin, DEU

**Keywords:** gout, arthritis, second metatarsophalangeal, joint, surgery, evacuation, gouty tophi

## Abstract

Gout is one of the most common diseases affecting men globally due to the spread of unhealthy dietary habits, kidney disease, and the use of diuretics. It is characterized as having monosodium crystals depositions in the synovial fluid, which causes an inflammatory response and painful joints. Most of the time, it can be found affecting the first metatarsophalangeal joint or other large joints in concurrence with other disorders. Non-steroidal anti-inflammatory drugs (NSAIDs), colchicine, and steroids have been known to suppress such events in most cases without the need for any surgical intervention. We present a case of a 44-years-old medically free healthy male who presented with a case of gouty arthritis in the second distal interphalangeal joint. Initially, colchicine treatment was given along with NSAIDs, but symptoms failed to subside. After a persistent increase of swelling and pain, the surgical evacuation was sought to reduce the pain and exclude any other causes of arthritis. Histopathology report confirmed the presence of monosodium urate crystals without any signs of infection. In conclusion, surgical intervention of gouty arthritis can be beneficial in cases of persistent pain and increasing rate of swelling despite the medicinal trial, especially in unusual cases of gouty arthritis such as gout of the second metatarsophalangeal joint.

## Introduction

Gout is defined as the deposition of monosodium urate crystals in the synovial fluids of the affected joint, which causes an inflammatory arthropathy and is considered the most prevalent joint inflammation in men [1, 2]. In most cases, it is found affecting the first metatarsophalangeal joint and can be commonly related to various metabolic disorders as well as obesity [3]. Diverse epidemiological factors for gout are found between different countries; however, its incidence and prevalence rate has increased since 1998, especially in developed countries. This is due to the increase in sedentary lifestyle habits with unhealthy dietary habits, obesity, chronic kidney disease, and diuretic usage in relation to hypertension. In the USA, the prevalence rate of gout was found to be 37.6/1000 compared to the UK of 4.7/1000, New Zealand 27/1000, and China 5.3/1000 [4]. Regardless, hyperuricemia or the elevation of uric acid has been identified as the main key in gout development. It is most commonly presented as an acute attack of synovitis after a prolonged time of asymptomatic hyperuricemia affecting the first metatarsophalangeal joint. The attacks are usually presented as a sudden onset of severe joint pain, reaching its most severity within the first 24 hours. The treatment approach to such attacks is non-steroidal anti-inflammatory drugs (NSAIDs), colchicine, or steroids. However, it can subside conservatively without treatment over two to three weeks duration [2]. We present here a case of gouty arthritis affecting the second distal interphalangeal joint.

## Case presentation

**Figure 1:**
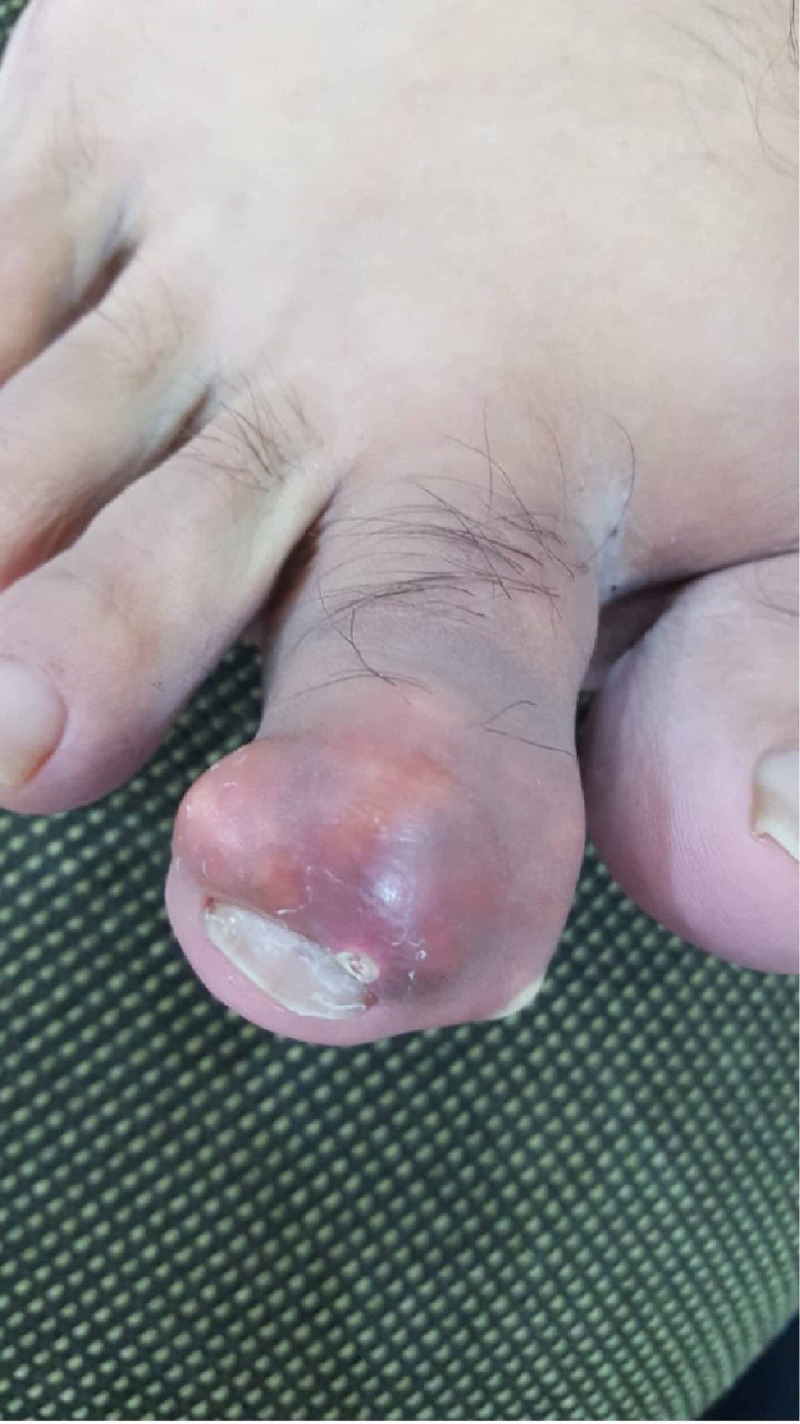
Initial presentation of the second toe showing swelling, redness, and white spot on the medial side with slight deformity

A 44-years-old male, medically free, presented to the orthopedics outpatient clinic complaining of a sudden severe painful swelling of the second toe with whitish spots for a duration of two days. There was no history of trauma, insect bites, rigorous exercises, or previous similar attacks. Regarding family history, the patient stated that there was a tendency in the family to develop gout and gouty arthritis; however, there were no chronic diseases present such as hypertension or kidney disease. Nutritionally, the patient did follow a healthy dietary pattern and was not obese. On inspection, an irregular swelling of the second distal interphalangeal joint was seen with redness and a large white spot on the medial side of the joint with noticeable slight joint deformity. On further examination, the swelling was hard, tender, and hot, measuring 1x2 cm. Furthermore, there was a limited range of motion with increasing tenderness and stiffness (Figure 1).

**Figure 2:**
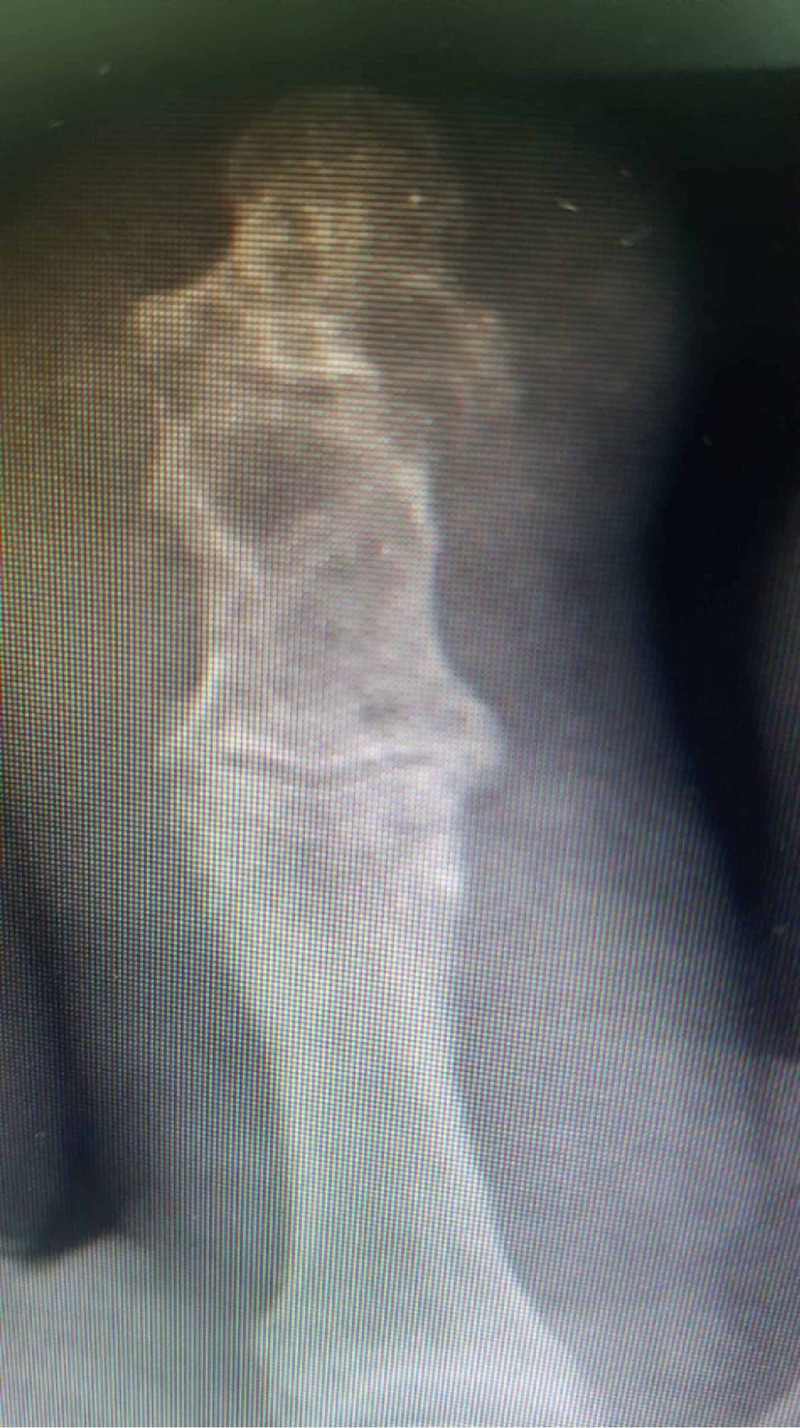
X-ray of the second toe on initial presentation showing no signs of osteomyelitis or bony destruction

Afterward, lab investigations included complete blood count, urea and electrolytes, urine analysis, erythrocyte sedimentation rate (ESR), and C-reactive protein (CRP) and were all within normal limits. Moreover, the rheumatoid factor was negative, but his uric acid was slightly elevated at 10.26 mg/dl (normal reference: 3.5-7.2 mg/dl). Next, an X-ray was done on the second toe showed signs of early erosions but did not show any signs of osteomyelitis or bony destruction (Figure 2).

**Figure 3:**
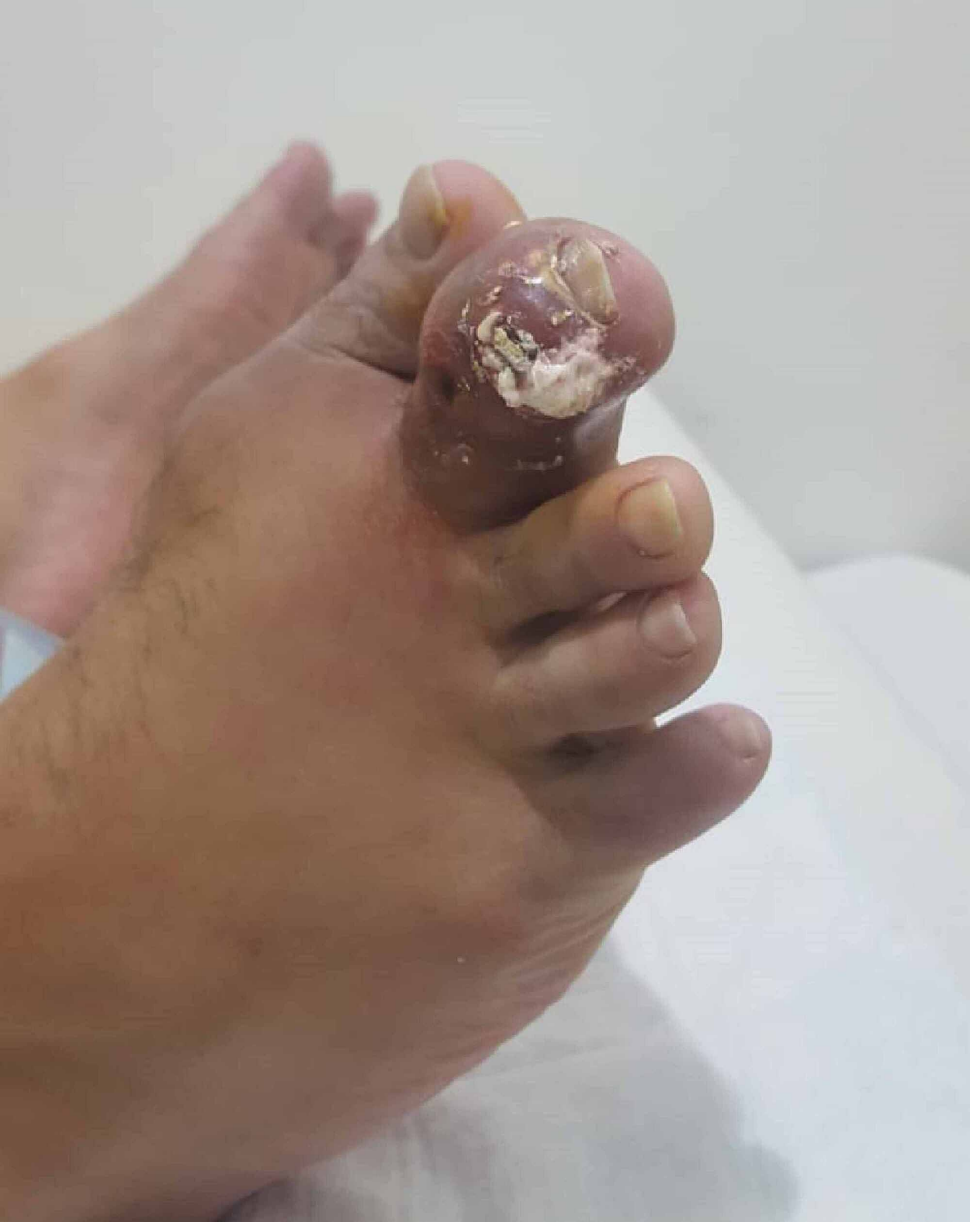
Increased swelling and discharge on the follow-up visit

His treatment plan was to start colchicine 500 microgram three times a day, NSAIDs (i.e., ibuprofen) 600 mg four times a day, and allopurinol 300 mg once a day for a duration of one week with further follow-up. Two weeks later, the patient returned to the outpatient clinic with increased swelling, pain, hotness, stiffness, and decreased ranged of motions, and according to him, he was fully compliant with the dosage and medication. Upon examination, increased swelling, as well as new white, odorless discharge oozing from the swelling were noticed (Figure 3).

**Figure 4:**
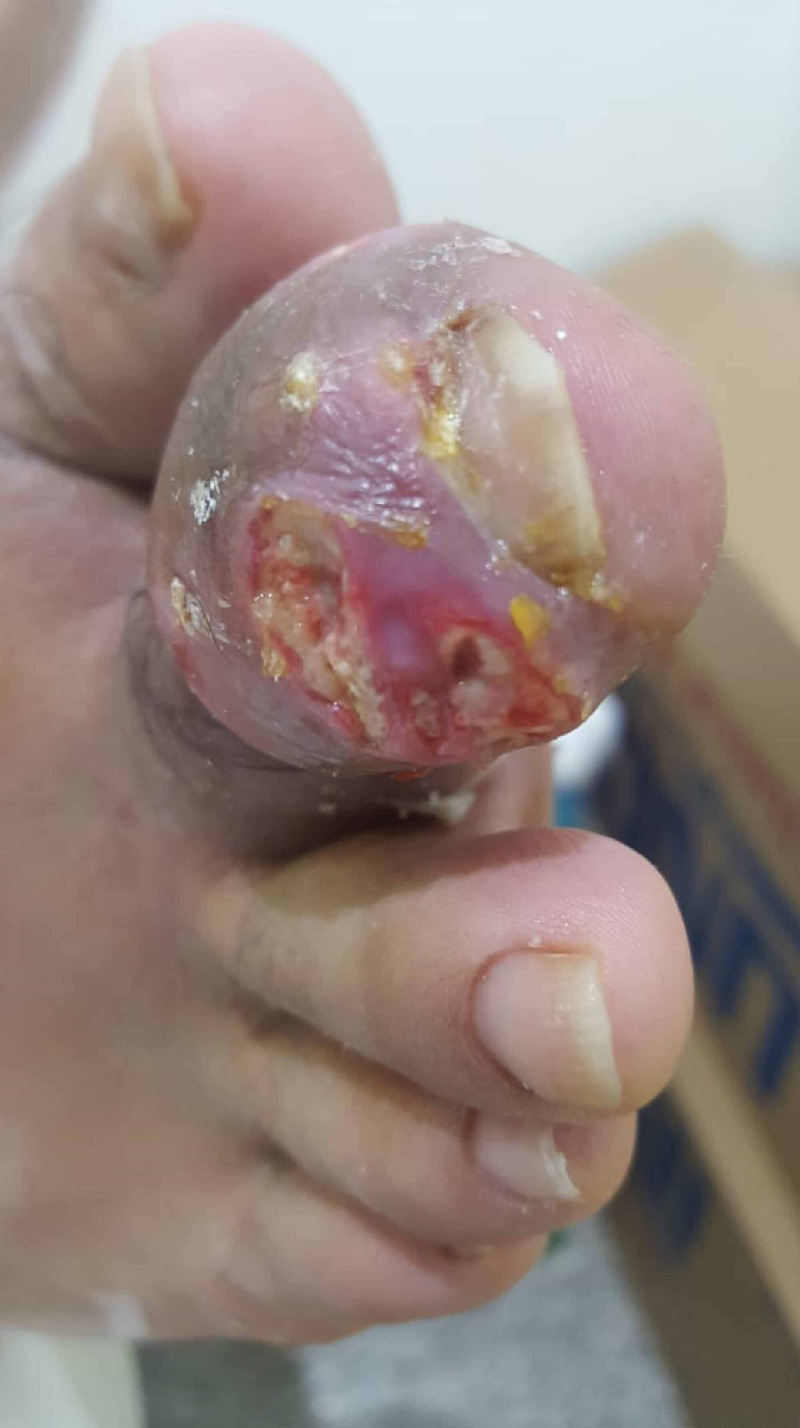
Evacuation performed by gentle pressure before complete surgical debridement

Evacuation by gentle pressure was performed under antiseptic measures and local anesthetic spray. Afterward, the patient was sent home with an appointment after two days for surgical evacuation to allow for the inflammation to subside. Two days later, surgical debridement was performed with the complete evacuation of the tophi under the local anesthetic digital block with daily dressing changes and regular follow-ups on weekly intervals (Figure 4). The discharge was sent to the histopathology lab and confirmed the presence of monosodium urate crystals.

**Figure 5:**
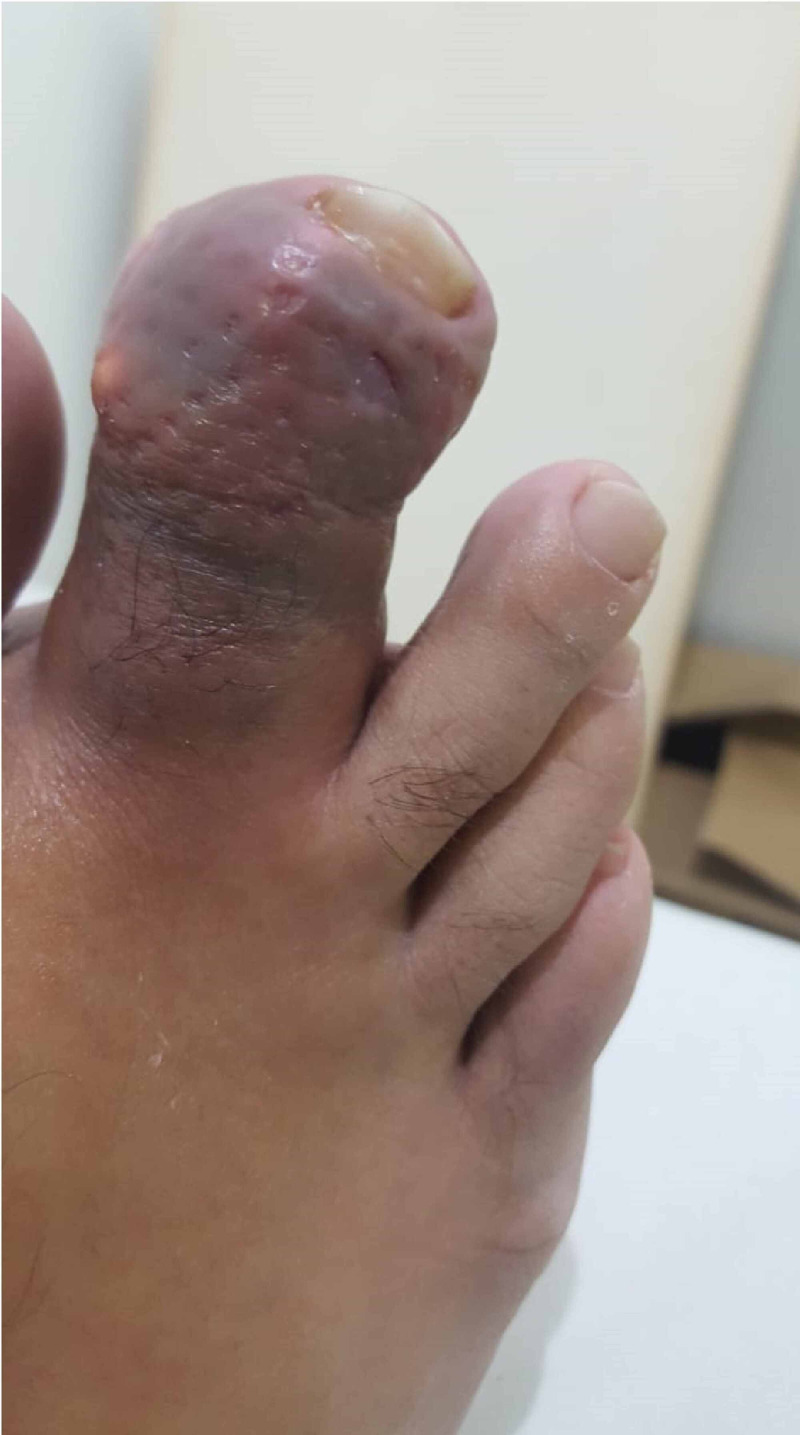
The second toe one month after surgical intervention

Over the period of follow up, the swelling started to subside, and the patient's pain and tenderness were much better by the second week after evacuation and debridement. He continued the same drugs, which were colchicine, NSAIDs, and allopurinol on the same dosage for the duration of follow-up, and by the end of the second month, the wound and the swelling was resolved to a great extent without the need for further surgical intervention (Figure 5). According to a phone follow-up, the patient did not report any flare-ups, and the pain subsided completely.

## Discussion

Hyperuricemia is one of the main causes of the deposition of monosodium urate in the joints leading to acute attacks of gouty arthritis. If not treated and left to resolve spontaneously, the patient usually enters an intercritical period, where further attacks occur with higher pain intensity, affecting different joints with a possibility to turn into polyarthritis in very rare incidents. However, the pathophysiology of hyperuricemia can either be secondary to dietary habits, kidney disease, or, as the case with our patient, might have a genetic/familial disposition factor as well [2]. It has been reported that in genetic cases of hyperuricemia, the association of the SLC2A9 gene with serum urea concentration has been found. Different variations of this gene might cause a tendency in some populations to increase the buildup of serum urea storage in the body by affecting the basolateral membrane of the renal collecting duct. Furthermore, the ABCG2 gene, which is responsible for the urate transfer activity, similarly has been shown to be affected by up to 53% of the Caucasian population leading to a lower renal urea excretion [5].

The incidence of gouty arthritis occurring in the second distal interphalangeal joint is extremely rare, with only one case being reported of a case of intermetatarsal space gouty tophi [6]. However, other presentations in different joints have been previously reported as secondary gouty arthritis to other disorders. For example, Reber et al. reported a case where they discovered the presence of tophi in the patella [7], while Jacobs et al. reported another case of a 77-year-old male with gout in the wrist most likely as a cause of medications [8]. In comparison to our case, the patient was medically free and was not taking any kind of over the counter drugs.

Even though hyperuricemia is a characteristic feature of gout, measuring the level of uric acid alone is not sufficient to conclude the diagnosis of tophi. The synovial fluid examination under the microscope for monosodium urate crystals is the gold standard to exclude any signs of possible infections. Therefore, the presentation of such rare incidence should always be diagnosed by synovial fluid analysis, so it would not be mistaken with other factors such as rheumatoid arthritis. If the synovial fluid analysis shows leukocytosis or low glucose levels, suspicion of infection in the sort of septic arthritis should be kept in mind [9].

Regarding radiological modalities used in diagnosing gouty arthritis, X-ray, which is the most used investigational modality initially, does not help diagnose early cases of bony destruction. Instead, it is useful in cases of chronic gout. As a result, it can help differentiate whether the case is in the acute or chronic phase of the disease. On the other hand, ultrasound started to gain more emphasis recently as it has both specific and nonspecific features of gouty arthritis. Specific features include a double contour sign, which is seen as an abnormal hyperechoic band over the superficial margin of the articular hyaline cartilage. Lastly, it is susceptible to having false-negative results. On the other hand, computed tomography has no role in diagnosing gouty arthritis as it is unable to detect inflammation or synovitis of the joint [9].

Colchicine is an alkaloid drug that acts as anti-inflammatory effects and is used in various diseases such as gouty arthritis, pericarditis, and familial Mediterranean fever. In gout, it has been recommended to start the initial dose of 500 micrograms thrice daily in the first 24 hours of the first flare-up. The use of high dosage versus low dosage treatment has been controversial. Even though one of the few clinical trials of the drug showed that a high dosage regiment can be superior to a low dose, most physicians prefer starting the low dosage to minimize the side effects of the drug, which can involve gastrointestinal, renal, and hepatic effects [10].

In a retrospective study in 2020, Kumar et al. studied 45 patients with surgically managed gouty arthritis. They reported that a small portion of patients (4%) underwent surgery for pain control, while 53% experienced delayed wound healing, and three patients (7%) required subsequent digital amputation [11]. In our case, surgical evacuation of tophi was done for pain relief as medical management failed to reduce the swelling or decrease the pain. Therefore, surgical management in such a unique and unusual presentation might prove beneficial in treating gouty arthritis.

## Conclusions

Gouty arthritis commonly affects the first metatarsophalangeal joint in men. The presence of tophi in the second interphalangeal joint is rare, and familial and genetic causes must be kept in mind as the main culprit, especially in patients who had no history of trauma or use of medication. Surgical debridement in the case of medication failure and pain relief should be considered in some cases of gouty arthritis.
